# Management of Chronic Musculoskeletal Disorders in the Workplace from the Perspective of Older Employees: A Mixed Methods Research Study

**DOI:** 10.3390/ijerph19159348

**Published:** 2022-07-30

**Authors:** Glykeria Skamagki, Christine Carpenter, Andrew King, Charlotte Wåhlin

**Affiliations:** 1 School of Sport, Exercise and Rehabilitation Sciences, Department of Physiotherapy, University of Birmingham, Birmingham B15 2TT, UK; 2 Department of Occupational Sciences and Occupational Therapy, University of British Columbia, Vancouver, BC V6T 1Z4, Canada; chris.carpenter@ubc.ca; 3 School of Nursing, Midwifery and Health, Department of Physiotherapy, Coventry University, Coventry CV1 5FB, UK; hsx471@coventry.ac.uk; 4 Occupational and Environmental Medicine Center, Department of Health, Medicine and Caring Sciences, Division of Prevention, Division of Rehabilitation and Community Medicine, Linköping University, SE-581 83 Linköping, Sweden; charlotte.wahlin@regionostergotland.se; 5 Unit of Intervention and Implementation Research, Institute for Environmental Medicine, Karolinska Institute, SE-171 77 Stockholm, Sweden

**Keywords:** chronic musculoskeletal disorders, ageing workforce, management, occupational health and safety, mixed methods research

## Abstract

(1) Background: This mixed methods research (MMR) study explored older employees’ experiences of chronic musculoskeletal disorders (CMSDs) in relation to their employment, their perspectives on managing these conditions in the workplace and the strategies used to facilitate and maintain their roles and responsibilities. The services offered to them were also identified. (2) Methods: A mixed methods exploratory sequential design was implemented. In the first qualitative phase, 16 semi-structured interviews gathered in-depth information from older employees. The findings informed the development of an online questionnaire in the survey phase, which was administered to older employees (*N* = 107). Both sets of findings were then integrated using a narrative joint display. (3) Results: The phenomena of presenteeism and leaveism were important components of employees’ strategies for managing their condition. The integrated findings highlighted the roles of employers, managers and social support in encouraging disclosure and supporting the management of CMSDs. The results also emphasised how self-management and professional health services are crucial for sustaining employability. (4) Conclusions: Current challenges call for employers to identify effective ways to support the ageing workforce and invest in training opportunities for managers and collaborative opportunities with healthcare professionals and other stakeholders. A flexible, empathetic and resourceful work environment is optimal for supporting sustained employability for an ageing workforce.

## 1. Introduction

Chronic musculoskeletal disorders (CMSDs) continue to be a leading cause of long-term pain and disability worldwide, affecting both the individual and society [1,2,3]. In 2017, The Global Burden of Diseases, Injuries, and Risk Factors Study (GBD) assessed the incidence and prevalence of 354 diseases and injuries and the number of years lived with disability (YLDs) in 195 countries and territories and found that, globally, CMSDs accounted for the second-highest number of years lived with disability [4]. These disorders include a range of inflammatory and degenerative conditions and syndromes that last more than 12 weeks [5,6] and share some common characteristics such as pain, stiffness and reduced mobility [7]. However, due to the intermittent nature and diverse symptoms, CMSDs may go unnoticed in the workplace as they may be “non-visible” [8].

In addition, the workforce has become remarkably diverse due to the integration of older employees and the increased number of women entering the labour force [9,10,11,12,13]. The prevalence of musculoskeletal conditions (including CMSDs) remains high in workers over the age of 50 in comparison to younger employees, regardless of the type of complaint [14]. The UK government has raised the statutory pension age (SPA) and delayed the pensionable age as a result of the increasing cost of providing pensions and services for the ageing population [15,16]. However, the movement towards sustained employability poses many challenges to the ageing population that may not be in line with the intentions of political reforms.

During the last decade, governmental and professional bodies in the UK have published reports that target the musculoskeletal health and wellbeing of older workers and discuss ways to extend working lives and improve employees’ work performance [17,18,19,20]. At the same time, a variety of models and recommendations have been developed aimed at shifting responsibility for health promotion to the employer [12,21,22,23,24]. However, it is still uncertain whether employers and managers are sufficiently informed or even motivated to support employees with CMSDs in staying healthy at work [25,26,27]. Exiting employment on health grounds may be a preferable and legitimate option for those with a CMSD. However, taking early retirement may not be a viable choice for many in the UK, as they will not be entitled to take their pension until the age of 67 or 68, depending on their birth year.

Different internal and external factors influence work (dis)ability, such as values, attitudes and legislative systems [28,29]. Therefore the degree of (dis)ability experienced by an ageing workforce is not only affected by the ageing process and the CMSD but also by the work context and other environmental factors, for example, the work demands and the environment confronting the employee [28,30]. In addition, studies to improve and manage musculoskeletal health in the workplace remain relatively overshadowed by those exploring prevention or return-to-work strategies [31,32,33,34,35,36]. Research has also highlighted the role of self-management programmes [37,38] and the importance of prescribed exercises for those with a CMSD [39,40].

In order to address the key issues identified in the literature, the design choices and implementation of this study were guided by an overarching aim and a number of related objectives. The aim was to explore older employees’ experiences of CMSDs in relation to their employment, their perspectives on managing these conditions in the workplace and the strategies used to facilitate and maintain their roles and responsibilities, and to identify what services had been offered to them at work.

## 2. Materials and Methods

**Figure 1:**
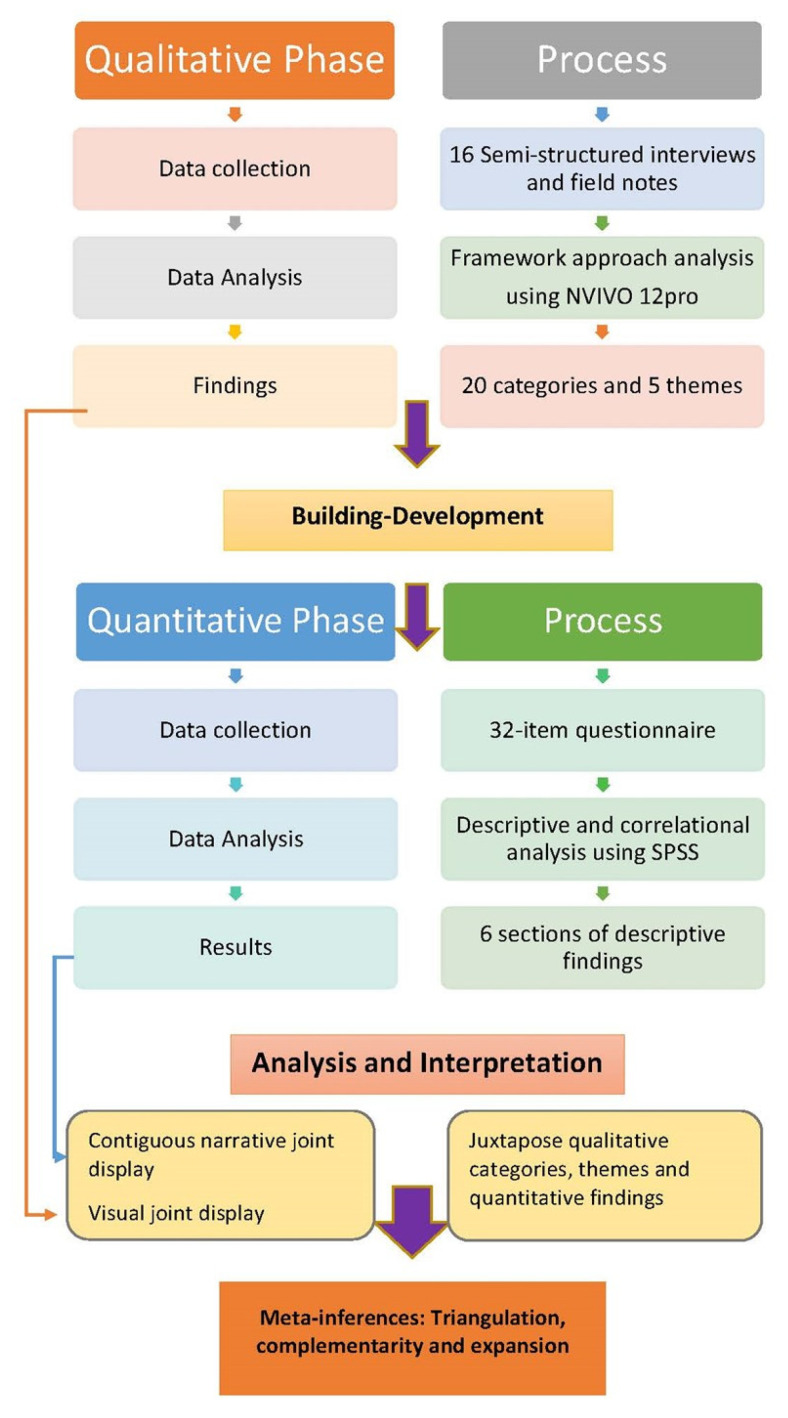
Overview of the exploratory sequential design. Note: in the framework approach, during the analysis process, codes are grouped into clusters around similar and interrelated ideas, called categories.

An exploratory sequential mixed methods research (MMR) design consisting of a qualitative and a quantitative phase (Figure 1) was chosen as the most appropriate for addressing the study aim. There continues to be considerable discussion about the philosophical foundation of MMR [41,42,43]; however, these authors generally agree that pragmatism provides a philosophical framework for designing and conducting MMR. Pragmatism, as a research paradigm, accepts that there can be single or multiple realities that are open to empirical inquiry. Thus, pragmatism focuses on what is practical and significant in the real world, which is more important than the abstract philosophies of the past [42,43,44]. This study was conducted in the West Midlands, UK. The West Midlands is one of the largest urban areas in the country, presenting a high incidence of CMSDs, and is considered to be representative of the working population of the country as a whole [45].

### 2.1. Using the Arena of Work Disability Prevention Model and the Work Ability House Model

**Figure 2:**
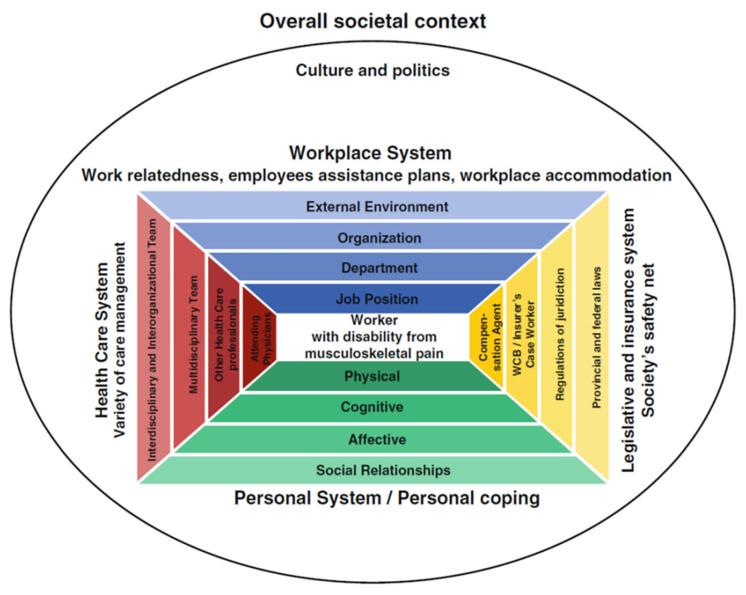
The arena of work disability model. Reprinted by permission from Springer Nature Customer Service Centre GmbH, Journal of Occupational Rehabilitation, Prevention of Work Disability Due to Musculoskeletal Disorders: The Challenge of Implementing Evidence, Loisel et al. [48].

Sustainable employability is connected to work ability and disability management [25,28,46,47]. The degree of (dis)ability experienced by an ageing workforce is not only affected by the ageing process, gender and the CMSDs experienced by employees but also by the work context and other environmental factors, for example work demands and the work environment confronting the employee [28,30]. The arena in work disability prevention model [48] and the work ability house model [49] provide a solid theoretical foundation that facilitates the exploration of sustainable employability in older workers, as they are built on communication and collaboration between the structures and the different systems influencing working life [29,30,48,49]. The arena of work disability prevention model (Figure 2) represents the worker with a disability as embedded in interacting individual, organisational and socio-political structures, e.g., personal factors, the workplace and the compensation system [50,51], which may influence the degree to which the disability impacts the individual in the workplace. It also highlights the impact that different stakeholders in each system can have on process decisions and worker support. The model guided this research in recognising how a diversity of factors associated with specific legal and cultural systems may influence an individual’s decision to participate in work.

**Figure 3:**
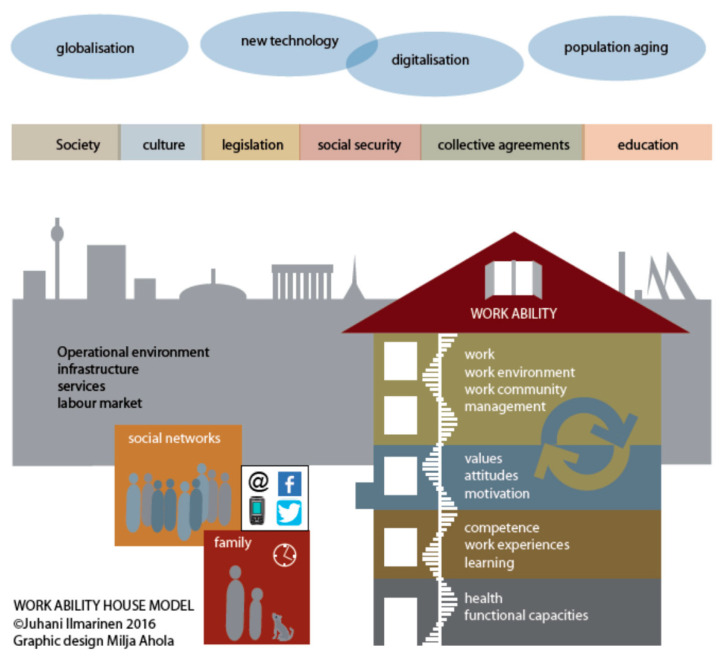
An illustration of the work ability house model taken from the latest published version [51]. This is an open access article distributed under the Creative Commons Attribution License which permits unrestricted use, distribution, and reproduction in any medium, provided the original work is properly cited.

The work ability house model (Figure 3) [49,52] focuses on the individual and on how a longer employment career can be supported and the quality of employment enhanced, by examining powerful relationships between contextual factors and an individual’s work and personal life. The work ability house model is “an evidence-based, comprehensive and systematic model for developing workplaces that facilitate better and longer worker careers” [49]. The model is represented by a house with four interacting floors. The three lower floors of the house relate to the employee’s resources: health and functional capacities (e.g., physical and mental), competence (e.g., training and knowledge) and values, attitudes and motivation (e.g., job security and finances). The fourth floor relates to aspects of work, the work community and leadership; for example, it may include the manager’s role and ability to provide resources. This model complements the arena of work disability model as it includes other divergent perspectives such as competence, health, other qualifications, family, occupational virtues, attitudes and values [47,53].

### 2.2. Ethical Approval

Both phases of this study were reviewed and approved by the Coventry University Research Ethics Committee. The research design followed the codes of ethics and conduct, and consent was gained from the participants and respondents who took part in the study. All necessary steps to preserve confidentiality and anonymity were taken.

### 2.3. Participants Selection

Participants in this study were employees over the age of 50 who were working in either private or public enterprises and who reported at least one CMSD (12 weeks or more) in any area of the body. Exclusion criteria for the study included individuals who were self-employed or part-time, employees who had applied for early retirement and those who had a specific pathological condition (e.g., tumours, infections) or an acute (as opposed to chronic) musculoskeletal disorder (Table 1).

### 2.4. Sampling Strategy for MMR

In the exploratory sequential design, the sample was different but it was drawn from the same population. This study aimed to gather in-depth information from employees over the age of 50 who had CMSDs and were employed in a variety of workplaces. As the purpose of the study was exploratory, a non-probability purposive sampling approach was chosen for both phases. Lavrakas and Battaglia ([54], p. 525) explain that: “purposive sampling is appropriate for the selection of small samples, from a limited geographic area or from a defined but restricted population where inference to the population is not the highest priority”.

#### 2.4.1. Sampling Strategy in Qualitative Phase

The qualitative phase employed a strategic approach to recruiting only participants who had the experiences and knowledge relevant to the research aim, i.e., in this study, older employees with a CMSD. Participant recruitment continued until rich data and data saturation had been achieved [55]. Data saturation, as a concept, has been generally accepted in qualitative research as a way of determining the quantity of data that should be gathered, e.g., how many interviews should be conducted. It is said to occur when little or no additional information related to the study topic is generated from the data collection process [56,57].

#### 2.4.2. Sampling Strategy in Quantitative Phase

In the survey phase of this study, a non-probability sampling technique was selected as the most appropriate method of accessing a cross section of the selected population in the West Midlands. There are four types of non-probability sampling to consider: quota, convenience, snowball and purposive sampling [55,58]. In this phase, it was not possible to acquire a list of potential participants that met the inclusion criteria and randomly send the questionnaire to them. Therefore, a purposive sampling approach was chosen.

### 2.5. Participant Recruitment

The first step in recruiting participants for both phases of the study was to identify representatives of a diverse number of large, medium-sized and/or small companies and to discuss the study with them. Permission to involve employees in data collection at these sites was sought from multiple individuals (described as “gatekeepers”) who worked in the human resources (HR) department or had a managerial role and were employed by large and medium organisations in the West Midlands to oversee the work sites.

#### 2.5.1. Qualitative Phase: Recruitment

In the qualitative phase, gatekeepers were contacted by an email in which the lead researcher introduced herself and provided information about the study and what it would entail for both the company and employees. The email also reassured gatekeepers about confidentiality and data protection processes. A participant information leaflet and an introductory letter were attached to the email. Finally, a phone call or a visit to the company’s site was arranged with those who replied positively, to discuss the study further and answer questions or provide clarification.

#### 2.5.2. Quantitative Phase: Recruitment

In the survey research component, the questionnaire link was distributed (in June 2019) to the previously identified “gatekeepers”. An email was sent to them with an information leaflet and an A4 poster advertisement that included a QR code and a website link, to enable potential respondents to access the online questionnaire. To increase the number of completed questionnaires, the gatekeepers received a follow-up message that was sent two weeks later, on the same day of the week and at the same time. The recruitment process for the survey phase was more challenging, as many of the companies that had previously agreed to participate responded negatively when they were asked to advertise the study to their employees. At this point, only 30 questionnaires had been returned, and the decision was taken to use social media and paid advertisements in the local newspaper to recruit more respondents. At the end of the third week, we had received 107 completed questionnaires. Five respondents were screened out as they did not meet the inclusion criteria.

### 2.6. Qualitative Phase: Data Collection

In the qualitative phase, face-to-face semi-structured interviews using a topic guide were chosen as the most appropriate method of collecting rich descriptions of the employees’ experiences. The interview guide was based on the findings of a quantitative systematic review and a qualitative meta-synthesis conducted by the first author as part of the requirements of her PhD programme and reviewed by the supervisory team. An online reflective blog and a diary were kept throughout the research process to enhance reflexivity. The first author also engaged in ongoing critical reflection on her values, attitudes and behaviours that derived from her experiences as a physiotherapist in Greece and the United Kingdom and from her personal background. These reflections were facilitated by a bracketing interview [59], which was conducted by a supervisor before the data collection began.

Two pilot interviews were conducted with volunteers who met the inclusion criteria, and they were recorded and transcribed. The transcripts were reviewed by members of the supervisory committee, enabling fine-tuning of the interview guide and the first author’s interview process and skills. The interviews were scheduled on a day and at a time convenient for each participant and lasted between 45 and 60 min. Consent was obtained in person by the interviewer. All the recorded interviews were transcribed verbatim, and the transcriptions were organised according to the participants’ pseudonyms and uploaded to NVivo 12 software. All memos and field notes were transferred to the software as expanded write-ups.

### 2.7. Qualitative Phase: Data Analysis

**Figure 4:**
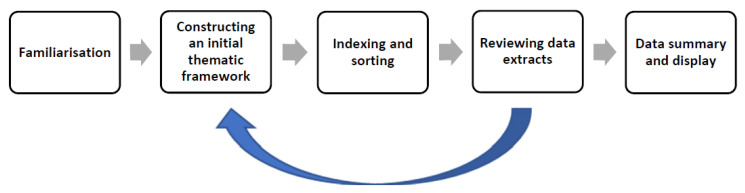
Framework analysis process [61].

In MMR, a “generic” qualitative analysis approach is commonly used. In order to provide structure to the analytic process a “framework approach” [60,61,62] developed by Spencer et al. [61] was used, comprising five intertwined stages (Figure 4). The analytic process began with the first author familiarising herself with the data by reading and re-reading the transcripts and then coding the data. The coding process was also reviewed by another author who is an expert in qualitative research. In total, 238 codes were created from the 16 interviews, and a coding matrix was created in NVivo 12 for each of them. Identifying codes and clustering codes into categories was aided by “mapping” exercises. Descriptions of each category were created, and categories with comparable meanings were then further grouped under broader “thematic” titles. The labels and descriptions used for the raw data were revisited and carefully examined to determine whether they accurately reflected the interview data and whether coded content needed to be moved to different categories. This process generated twenty categories and a final five themes. The qualitative-phase findings were used to develop a questionnaire for the survey research phase.

### 2.8. Quantitative Phase: Data Collection

In the second phase of this study, a descriptive cross-sectional survey approach was used to systematically collect quantifiable data in a single data collection period, identify the characteristics and other variables of employees over the age of 50 with a CMSD and create associations between these variables. A scoping review, defined by Grant and Booth [63] as “a preliminary assessment of potential size and scope of the available research literature that aims to identify the nature and extent of research evidence”, was conducted to identify established and validated questionnaires that could be used in, or adapted for, the survey phase of this MMR study. Two questionnaires, the Health and Employment After Fifty questionnaire [64] and the Nordic Musculoskeletal Questionnaire [65], examined the impact of general health on employment; however, they primarily focused on the prevalence of MSK issues or compared the impact of MSK problems in specific areas of the body using a body chart. Neither questionnaire specifically addressed issues related to managing CMSDs in the workplace and the strategies used by employees. Consequently, we decided to construct a new questionnaire, using Johnson and Christensen’s ([57], p. 209) principles to guide the process. One item from the Work Ability Index [66] was included with permission.

The final questionnaire (Supplementary Materials) consisted of 36 questions organised into 6 sections designed to comprehensively explore the management of CMSDs by older employees (Table 2). This online survey tool was designed for academic research, education and public sector organisations and is fully compliant with UK data protection laws and the General Data Protection Regulation (EU) 2016/679.

The online questionnaire generated numerical (nominal and ordinal) data which were coded and analysed using descriptive statistics and SPSS 21 software. Non-parametric methods (charts or graphs) were employed to report the data. The chi-squared test and Spearman’s rho (ρ, also signified by rs) correlation coefficient were chosen as non-parametric tests to measure the strength and association between two ordinal variables [67]. The data generated from the single-forced-response questions are presented as percentages, with the exception of the data acquired from multiple-response questions which are presented as the number of respondents.

A total of 112 questionnaires were received, and 107 questionnaires were included in the final database. If respondents did not answer a forced-response question, they were not able to advance to the next page and were required to close their Internet browser and withdraw. In this way, the online surveys tool ensured that there were no spoiled questionnaires. A total of 5 questionnaires were excluded from the data analysis through routing questions related to the age range (below 50 years old), employment status (self-employed or retired) and stage of the MSD (<3 months), which identified respondents who did not fulfil the inclusion criteria.

### 2.9. The Integration Process in an Exploratory Sequential MMR Study

**Figure 5:**
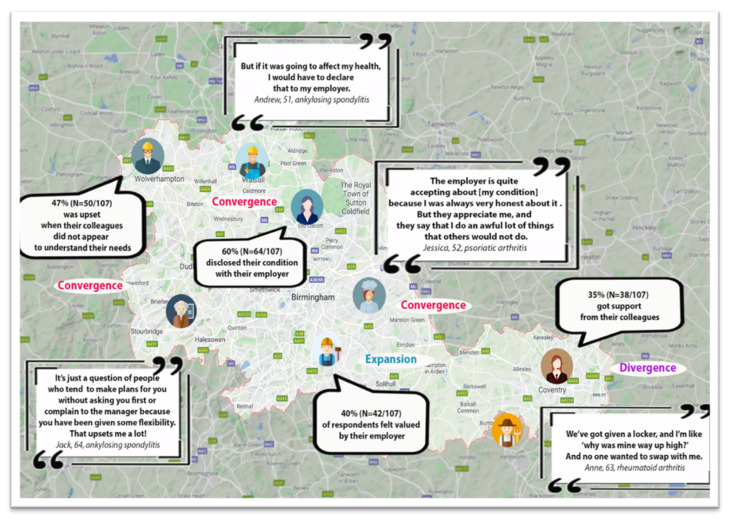
A visual joint display of the integrated findings related to the theme “social support and disclosure”. Note: graphics were used to represent employment roles discussed by the study participants, and the related survey findings and examples of illustrative qualitative quotes are presented in bubbles.

Integration of the different components of a MMR study is an essential characteristic of MMR [68,69] that enables equal priority to be assigned to all the study components. Through such integration, the component findings can be juxtaposed in order to align and expand the study findings. Integration in this study occurred at the methods and the interpretative analytic levels [42]. A narrative joint display table (Table 3) and a visual representation using geocoding (Figure 5) illustrate the integration process [42,70]. Although these are not essential, they can help the reader to understand the analytic process and overall findings. Meta-inferences (overall conclusions that are developed through an integration of the inferences obtained from the qualitative and quantitative strands of a mixed methods study) [43] were created from the integrated qualitative and survey findings, providing new insights that addressed the study aim and objectives. The overarching themes are identified and discussed in Section 4.

## 3. Results

A total of 16 employees, comprising 4 males and 12 females, were recruited over 3 months for the qualitative phase, where 12 participants were performing professional, managerial or administrative work and the other 3 participants worked in skilled manufacturing, construction and technical installation. In addition, 107 employees over the age of 50 completed the online self-administered questionnaire.

Most of the respondents worked in education (20%), and others were employed in healthcare (14%) and manufacturing (14%). A total of 65% of the respondents were employed in large companies (more than 250 employees), 10% in companies with between 50 and 250 employees and 27% in small companies employing under 50 people. Finally, 40% of the sample reported chronic low back pain, 27% an inflammatory MSK condition, 14% osteoarthritis, 11% upper-limb disorders and 4% chronic neck conditions. The socio-demographic characteristics of the interview participants and survey respondents are presented in Table 4 and Table 5.

In the following section, the findings from the two phases are presented using a “weaving” approach [42]. The interview quotations highlight the identified qualitative themes, while percentages in brackets are used to indicate the proportion of questionnaire respondents who expressed agreement or disagreement with a particular interview theme or else complemented and expanded upon it. The five main qualitative themes and the survey findings together illustrate multifactorial linkages between experiences, attitudes and the management of CMSDs within the ageing workforce. The final categories that contributed to each qualitative theme are given in Table 6. The qualitative themes drive the results section below. Themes are highlighted with a bold font.

**Impact on Wellness**, the first qualitative theme, focused on how CMSDs have affected different areas of the participants’ lives, including work, social life and family. All participants agreed that their condition, the changes in the state pension age and other related factors in the work environment had caused them extra mental stress with associated emotional impacts. In the interviews, employees explained that they were motivated to attend work and therefore their health was important to them. Sally explained that her arthritis affected her ability to climb stairs:

“A couple of steps I can manage, but if it is a lot of steps, it just takes me ages to get to them. But you know, sometimes I’m all right. I can do it.”

These findings were further illustrated by the questionnaire results, where 53% of the respondents scored low on the work ability questionnaire item and 60% reported that the CMSD interfered “quite a lot/extremely” with their ability to work effectively during the past six months. Similarly, 80% reported that it was “very” to “extremely important” to perform well within their job, and 92% indicated the importance of mental and emotional wellbeing.

The **Strategies and Facilitators** that participants themselves used to manage CMSDs at work were explored in the second theme. Participants discussed how they self-managed their condition using strategies such as monitoring their sitting time or losing weight. Respondents in the survey phase also used strategies to maintain their health; for example, 25% monitored their physical activity, 66% took regular breaks while at work, 73% indicated that they managed the condition with appropriate medication and 33% made lifestyle changes. Laura described how she became more active and efficient in the workplace by using a smartwatch:

“So, it tells me, you know, come on you have got only done so many steps this hour, so get up and move.”

Some participants in the qualitative phase suggested that employers also offered strategies that were integrated into the workplace policies and described how their organisation referred them to physiotherapy services, a gym or both. The strategies chosen from those listed in the questionnaire were similar; for example, 17% were offered an online occupational health assessment, 19% were referred to the on-site physiotherapy clinic and 26% were offered flexible hours. Jessica explained the difference between her previous and current employers and their attitude towards flexible working hours:

“My previous job offered me ‘Flexi-time’, which meant that If I couldn’t always be in at 8:00 [due to a bad night with arthritis], I could stay and work from home and attend the day later. That was really useful and beneficial.”

Participants in the qualitative phase felt that their manager or colleagues were supportive and empathetic when they saw them struggling and would help them with particular tasks that they found difficult. Those who had themselves experienced a musculoskeletal problem were perceived as being more understanding. The survey results were similar, indicating that 35% of the respondents received support from their colleagues, 26% felt happy with the support they received from their organisation and 42% indicated that they were informed about the strategies available to them. Similarly, respondents who reported a supportive line manager were also more aware of the relevant strategies (r (105) = 0.365, *p* < 0.001) and felt satisfied with what was offered to them (r (105) = 0.540, *p* < 0.001). Annette described how those colleagues who had had a similar experience or injury were more likely to empathise with her and be helpful:

“My colleague had a hip operation, so you know… him being through his operation and me with my arthritis. So, we have an understanding, if you know what I mean.”

Lastly, participants discussed in some detail how they managed the condition outside the workplace. Participants hoped that they would remain healthy, because they wanted both to enjoy personal activities and to continue to perform well at work. The questionnaire results demonstrated that some employees were highly motivated to manage their condition in both their professional and personal lives. For example, 79% had consulted a physiotherapist privately and 70% indicated that they had used passive strategies such as massage therapy services or acupuncture, while 49% preferred to exercise at the gym or go swimming. Annette discussed how her visit to a physiotherapist helped her to manage her arthritis better:

“My physiotherapist is very good. She listens, she understands, and I am happy with the treatment that I have had and the exercises of course.”

In the third qualitative theme, **Perceived Barriers to Management of a CSMD**, participants identified some aspects of their workplace environment as obstacles, for example buildings without elevators or with cold rooms. They also felt that the set-up of their workstation was not “fit for purpose”, for example a “hot-desking” policy. Similarly, 54% of respondents in the survey phase reported that their workstation needed alterations to accommodate their needs. Nicky discussed the use of the office chairs provided at work and how uncomfortable they were:

“We are all together in a big open plan office and they gave us those awful chairs. They were awful! I mean I literally couldn’t sit for longer than half an hour and I find I’m struggling.”

Participants highlighted that they did not have sufficient information about the services offered by their workplace and that accessing an OHS or other work-related support service was time-consuming. There was a significant positive relationship between a supportive employer, employees’ awareness of supportive strategies in the workplace (r (105) = 0.512, *p* < 0.001) and the levels of satisfaction with the strategies offered (r (105) = 0.690, *p* < 0.001). The survey results also indicated that 42% of the respondents had not discussed their needs with the OHS and 32% did not have access to an OHS or to other healthcare professionals in the workplace. As Kathryn suggested:

“I haven’t been asked anything and nobody sat down with me and said OK what is your condition, how bad is it, does it affect your work?”

Some participants described their colleagues as mostly unsupportive, and others felt that their colleagues did not recognise or acknowledge the condition and its impact on their working life. This was particularly the case when the CMSD was not obviously “visible”. Participants suggested that people at work were not well informed about CMSDs and that they did not understand how they can affect a person’s life. Quantitative results suggested that 22% of the respondents almost never received support from their colleagues and 35% declared that colleagues did not understand or recognise the impact of a CMSD at work. In addition, 60% were upset when their colleagues did not appear to understand their needs, and 56% reported that people were not well educated about CMSDs. Claire, in trying to explain why her colleagues were unsupportive, said:

“I just think people filter it out… people say to me now, ‘oh, you’ve hurt your leg’, because I’m in sandals and they can see that I’ve got a stocking and I’m thinking, no I’ve told you several times.”

Furthermore, participants in the qualitative phase identified barriers that they associated with management teams in both their current and previous employment. Some of them felt that smaller organisations did not have the resources to support their needs, and others thought that it depended on how much the company valued the individual. Some participants stated that their employers did not facilitate access to health care professionals. The survey results suggested that 24% of the respondents had no support in managing the CMSD in the workplace and 25% believed that their line manager or employer did not actually recognise or understand their condition. The correlational analysis showed a negative correlation between employees working with pain or discomfort and the support they received from their employer (r (105) = −0.360, *p* < 0.001), their line manager (r (105) = −0.195, *p* < 0.001) and their colleagues (r (105) = −0.273, *p* < 0.001). As Jessica said:

“I think the managers are under a lot of pressure and I don’t think that they always have an awful lot of capacity to deal with people that can’t just fit into a standard box for them.”

Lastly, participants spoke about external factors such as family roles, bureaucracy and waiting times in the National Healthcare System (NHS). All employees discussed their experiences of obstacles related to the NHS and how this affected the management of their condition. Some felt that the system was slow to respond to their health needs and that the waiting time for an appointment at the hospital was long. In the survey phase, 55% indicated that they did not have timely access to healthcare professionals through the NHS. Only 24% of the questionnaire respondents were happy with the support they received from the NHS, whereas 69% recognised that managing the condition in the workplace could potentially reduce the burden on the NHS.

The fourth theme, **Employees’ Approach to Living with a CMSD**, explored how the employees’ attitudes and experiences influenced the way they managed the condition. There was considerable diversity in the participants’ experiences. Participants discussed in some detail their work ethic and their ability to work over an extended time. They did not want to be perceived as different from other employees, and they shared their experiences of consistently needing to demonstrate a good work ethic. Thus, most of the participants wanted to stay at work, even on the days they did not feel particularly well. Others described taking annual leave as a response to strict organisational absence policies or as a strategy by which they hid their symptoms from colleagues and managers. Similarly, most of the respondents in the survey phase (73%) indicated that they worked with ongoing pain or discomfort, and 74% reported that they would remain at work even on the days they felt unwell. Only 20% reported that they would ask their colleagues to help them with a task if they were unable to perform well, and just 2% indicated that they would take sick leave when they were not feeling well. The correlational analysis showed that respondents who stayed at work on the days they did not feel well would not consider taking sick leave (r (105) = 0.445, *p* < 0.001). Sally suggested that people with CMSDs did not take sick leave as readily as their co-workers. As she said:

“Yes, I would work with pain. Because it’s been a feature of my life that I’ve just got used to having, you know… So, for me, you know to have a day that is, ah, the back’s a bit sore means so what?”

Participants felt that some of the strategies offered in the workplace were helpful, but most of them discussed the burden of needing to constantly self-manage and take the responsibility for implementing coping strategies. Some participants found coping with the condition and their job responsibilities challenging, especially if the work expectations were quite high and job-related tasks were complex or difficult. Employees in the survey phase reported that the various strategies offered in the workplace were effective and important to them; for example, 30% (*N* = 8/28) of the respondents perceived flexible working hours as an effective and an important strategy (35%, *N* = 9/28). Similarly, 34% (*N* = 7/21) found working from home effective, and 20% (*N* = 7/21) found it important, whereas only 5% (*N* = 1/18) perceived online occupational health assessment as effective and important. Overall, only 21% were satisfied with the strategies offered them, and only 37% were confident that they could manage their condition well without specific workplace support. Options are necessary to accommodate the different needs of employees, particularly those with a CSMD, but this imperative is not reflected in the study findings. For example, the retail industry primarily offered only the option of flexible working schedules.

Both interview participants and survey respondents highlighted the relentless nature of being required to self-manage and take the responsibility for implementing coping strategies. A total of 94% of the survey respondents declared that self-management was very important in maintaining their health. As Jessica said:

“It’s fair to expect people to support you but you have to take responsibility for managing your own health and wellbeing before other people can help you do that.”

However, only 39% of employees felt they could find the time needed to effectively self-manage their chronic condition. There was a medium correlation between those respondents who felt depressed and those who did not feel confident in their ability to effectively manage the CMSD (r (105) = −0.322, *p* < 0.01).

An interesting aspect of this theme was how employees expressed both negative and positive attitudes about disclosing their condition to their employer and work colleagues. Some discussed negative experiences related to previous employment experience which influenced their decision not to disclose their CMSD to their current employer. For example, although 67% disclosed the condition to their line manager and 60% to their employer, only 36% felt confident enough to discuss their needs with them. There was a significant correlation between employees with a chronic inflammatory condition and the decision to stay at work when feeling unwell (r (105) = 0.512, *p* < 0.001). The results also suggested that 63% of the respondents felt confident to discuss their CMSD with their colleagues after disclosure, but only 20% indicated that they had discussed the CMSD with their supervisor or line manager. Jack explained his perception that people who could not manage their conditions in the workplace would be easily replaced:

“People need to manage their conditions. It’s this animal instinct, where in the world of animals one is injured, one is a predator. The injured animal will always try to walk normally to convey to the predator that they’re not injured.”

The final qualitative theme, **Emotions and Beliefs about Future Employment and Retirement**, exposed how some participants were worried about their current and future employment and the consequences they might face due to their CMSD. Others expressed their fear of taking sick leave because they had experienced negative outcomes with past employers or had been criticised for aspects of their work related to the chronic condition. Similarly, 50% of the survey respondents felt worried about future employment due to their condition, and only 29% of the respondents felt confident that they could work until the retirement age. As Jessica explained:

“It worries me because it’s a progressive condition and it doesn’t matter what I do. I can stay as well as I can for as long as I can but there will come a point that it will become harder for me to stay mobile and I don’t know whether that’s going to be when I’m 62, 72. And how am I going to work?”

Most participants described how they were motivated to work for more years rather than take early retirement, as they either enjoyed their job, felt too young to give up or valued the mental challenge offered by work. Male participants were well informed about the changes to retirement, but they were not happy with the changes to pension age, which in their view were implemented by the government when the country’s economy was unstable. Female participants were concerned about retirement and were upset and angry about the proposed changes. They felt deceived, as the SPA had increased multiple times in the past few years and the choices they had made about their future and retirement had been affected. Furthermore, women were stressed about family responsibilities that affected their health, such as caring for elderly parents and older spouses with increasing health problems, looking after grandchildren or financially supporting their children who were studying or were low on the property ladder. The survey results suggested that 62% of the respondents were adequately informed about the pension age changes but only 47% felt confident to manage their finances until the new retirement age. Claire explained the difficulties ahead:

“And I don’t know what I will do if I cannot work, I’m hoping to make it till 60 and then I’ll review things. But I am still helping my daughter financially and I am also looking after my mum you see.”

Several participants discussed the benefits and disadvantages of staying at work until the age of 67. Some participants reflected on the strategies they had chosen for a healthy retirement and explained that maintaining their health would be their primary plan for a successful retirement. However, others felt that the changes to the retirement age would negatively impact the ability of people with chronic conditions to take or enjoy retirement. Those who did not foresee the changes in the retirement age and could not afford an early retirement were upset and afraid about their future health and their ability to keep working. The survey results suggested that 78% of the respondents felt that governmental changes would impact on their ability to enjoy retirement, but only 40% of them had a plan for how to manage their condition until that time. There was a strong negative correlation between those who were worried about their future employment and those who felt they could manage their finances well (r (105) = −0.506, *p* < 0.01). Lastly, there was a strong positive correlation between respondents who had a management plan in place and those who believed that they could work until their SPA (r (105) = 0.405, *p* < 0.001). For example, Maria had a plan:

“So, I will be taking what they call “phased retirement” which is the ideal thing for me. I could go down to 2 days; I can go down one more day before I actually fully retire.”

## 4. Discussion

In the section below, the integrated findings are discussed and positioned within the current research literature and presented under the newly emerged themes. Based on the study findings, the work ability house model [49] and the arena of work disability model [48] provided a solid theoretical foundation that guided the data collection and analysis phases of this study and the integration of the findings. These models contributed to a more comprehensive understanding of the management of CMSDs at work, provided a structured outline of the different influencing systems (e.g., socio-political) and acknowledged the key stakeholders involved in the management of CMSDs for the ageing workforce. As a result of integrating the findings, a number of meta-inferences were generated. These are discussed under the following seven headings and in relation to the current related literature.

### 4.1. Older Employees with CMSDs Face Uncertainty

This study showed that flare-ups and other common symptoms affected labour activities and created uncertainty about employees’ ability to fulfil their job requirements. Colleagues who had no exposure to, or knowledge of, chronic conditions were often unsupportive even on the days a co-worker experienced a flare-up with more visible symptoms. Findings illustrated that a number of employees were anxious or depressed due to employment uncertainty and reduced work performance, rather than the condition itself. Previous research has shown that it is important to consider the extent to which mental health conditions impact the experience of chronic pain and vice versa [71,72]. The confounding factors that affect relationships between the experience of managing a CMSD, mental health issues and sick leave should be explored.

### 4.2. Social Support for the Disclosure and Management of CMSDs

The integrated findings suggest that interpersonal relationships between older employees and their work colleagues can be negatively affected by lack of understanding of the condition and lack of empathy. The perceived lack of support and colleagues’ attitudes towards the CMSD affected employees’ decisions about whether to disclose the condition and revealed concerns about avoiding gossip and the scepticism of others. Older employees trusted their work abilities, valued their job role and were determined and motivated to perform well at work. These findings are supported by Smith and Brunner’s [73] study, which revealed that organisational culture and relational considerations may shape the environment for or against disclosure. The authors suggested that building trust and educating others about health conditions could positively influence disclosure. Therefore, the relationship between social support and empathy in the workplace should be explored, as developing these skills will allow co-workers to support those with CMSDs.

### 4.3. Presenteeism: Why Do Employees Come to Work When Unwell?

Employees in our study were motivated by a strong work ethic to stay at work even when they felt unwell, as they considered this to be “normal” behaviour. This phenomenon is represented in the literature as sickness *presenteeism*. The different intrinsic and extrinsic factors that are described in the literature as “driving” older workers to presenteeism [74,75,76] are reflected in the integrated findings. The findings also highlighted the fact that presenteeism was significantly higher for employees who felt ignored by the management team due to, for example, poor communication, a lack of sick pay or a culture of working long hours. In these situations, employees lost confidence in the employer’s or manager’s ability to make substantive changes in the work environment and to develop a management plan with them. Similar issues were identified in previous qualitative [77,78,79,80] and quantitative studies [81,82,83] that explored communication issues between the members of the management team and employees with CMSDs. Because presenteeism is highly responsive to the relationship between the individual and their work environment, it is important to understand how these factors influence decisions and to explore ways to moderate their effects on musculoskeletal health and performance.

This study adds to the association between disclosure, discrimination and presenteeism. Those employees who did not disclose their CMSDs were often vulnerable to prejudice due to disbelief or to judgemental comments about their work abilities. Research has highlighted the fact that employees with chronic diseases are vulnerable to discrimination. They are often required to cope not only with the negative effects of the condition but also with the negative attitudes of colleagues and managers [84,85]. As discrimination can be motivated by prejudice [86], employers need to acknowledge that stereotypes exist and that the threat of being stereotyped may have a negative effect on the older employee. Therefore, raising awareness in the workplace of the impact of working with a CMSD may benefit the ageing workforce and create a proactive work environment that discourages judgemental attitudes.

### 4.4. Presenteeism in Employees with Chronic Inflammatory Disorders (CIDs)

This study reveals the importance of supporting subgroups of the ageing workforce, especially those with CIDs. These employees were aware of the progressive nature of their disease and had experienced intermittent flare-ups. However, the findings indicated that they did not use their sick leave, even when their ability to work was affected, as they were concerned about disciplinary actions after reaching their maximum sick-leave allowance. Absenteeism can be a significant threat to productivity, staffing, costs and employee morale [87,88]. Employers can discourage frequent or long-term absence from work by implementing strict sickness absence control procedures [75,89]. A causative relationship between the specific effects of CIDs and presenteeism has not been established, but there is some evidence to suggest that these conditions influence presenteeism behaviour [90,91,92].

### 4.5. The Phenomenon of Leaveism

This study contributes to and expands knowledge about the phenomenon of *leaveism.* The integrated findings revealed that employees frequently chose to use their annual leave as a strategy to manage their CMSD. Leaveism may offer an explanation for these unexpected consequences of organisational policy and culture. Previous studies [93,94] also indicated that employees used annual leave, time in lieu and other leave entitlement schemes instead of their sick leave, taking time off when not feeling well or when they had nearly reached their maximum sick-leave allowance. There is a small but growing interest in exploring the impact of leaveism on employees’ physical and mental health. However, to date, only one study [95] appears to have directly addressed the issue of leaveism and discussed how research could contribute to understanding employees’ experiences of leaveism in the workplace. As absence policies and excessive workload may have an impact on the wellbeing and the work-life balance of employees with CMSDs, it is important for both employers and employees to become aware of this phenomenon. In contrast to the phenomenon of presenteeism, leaveism has had, to date, limited scholarly or research attention, and further research is required.

### 4.6. The Impact of State Pension Age Changes on Retirement

This study adds to knowledge about the impact CMSDs have on early retirement. The findings revealed the uncertainty and frustration older employees felt about the pension age reforms and how these were impacting their ability to enjoy life up to and after retirement. Health is a major factor influencing the decision to take early retirement [96], and many employees expressed concerns about managing more than one chronic condition and their ability to work until their new official retirement date. Employers need to understand that SPA changes will impact older employees, who may find it hard to meet the demands of their work, particularly if their health deteriorates or if they wish to exit employment in order to pursue other interests [97].

Similarly, the integrated findings illustrated how financial considerations influenced employee’s decisions about early retirement or remaining at work. Early retirement was not possible for those who had not been able to acquire sufficient financial resources. The qualitative findings helped to elucidate the problem that older employees who had reduced work ability were forced to stay in the job due to extrinsic factors such as economic dependency related to supporting children or repaying bank loans or mortgages, or because inadequate retirement plans had been made. Early retirement could cause financial stress, income loss and forced decisions that could potentially impact other family members [98]. It is important that employers support older employees with CMSDs in staying active and productive by developing and implementing management practices that will enhance the sustained employability of these employees.

### 4.7. The Influence of Gender on the Transition to Retirement

The study adds to knowledge about the difficulties women face in managing CMSDs at work. The integrated findings illustrated that women questioned their ability to make the last-minute adjustments required by the new retirement age. Planning for retirement often requires consideration over years about family, work, savings, pensions and early retirement options. Although most of the female participants indicated that they had been informed about the SPA changes, it was less clear to them how they could acquire sufficient financial resources to support their plans. Women more commonly compromise their career when the needs of children and other family members compete with work [99,100,101]. These changes in working pattern can become a challenge, as they may decrease financial capacity and pension contributions and, in reality, limit the access of women to early retirement [97,102,103]. As the Pensions Policy Institute Report [104] indicated, women are still paid on average 18% less than men in all occupations and, as a result, they accumulate only one third of the pension that a man acquires by the age of 60.

Findings indicated that assuming caring roles within the family affected an individual’s health and the quality of their personal lives. These factors contributed to a sense of loss of control in both personal and working lives. The Office for National Statistics report [105] suggested that, compared to men, women who had already adapted their life to look after children might be more likely to, or be expected to, adopt other caring roles such as looking after grandchildren or caring for their parents. These responsibilities could be particularly challenging for the older female worker, and these statistics should be considered when developing governmental and organisational policies. Policies aimed at extending working lives should take into account the caring responsibilities of employees over 50 years old.

### 4.8. Roles and Responsibilities of Employers in Supporting Employees

The findings highlighted the employer’s key role and responsibilities in helping older employees with CMSDs in the workplace. However, the strategies offered are subject to the size and the type of the industry, the job role, the organisational policies and acknowledgement of the impact of CMSDs at work. The integrated findings showed that most employers did not signpost employees to relevant services, even though national guidelines [12,106] encourage companies to consult healthcare professionals or engage occupational health services. A possible explanation could be that employers do not have a clear understanding of the OHS role. Another could be that some employers are hesitant to invest in resources, due to the perceived expense [107,108]. The feasibility of extending working lives depends to some extent on the employer’s approach to implementing governmental policies and providing individualised support to the older employee. Despite the importance of maintaining the health of the ageing workforce, there is still no legal requirement for employers to provide ergonomic assessments in the workplace or vocational rehabilitation.

National bodies and independent organisations [12,109,110] have published recommendations and guidance to empower employers to support the workforce in seeking professional advice. These guidelines are based on the best available evidence and have been collated by researchers, experts in the field and other stakeholders [111]. Our findings suggest that the employers involved were either unaware of the available schemes of support or did not understand how to apply the guidelines in the workplace. It is recognised that limited prescriptive information is provided to guide employers’ in using the published recommendations [107,112,113]. In addition, translating evidence-based practice (EBP) from the scientific environment to a practice-based context is a challenging task, due to the different perspectives of stakeholders, policymakers and researchers [114,115]. Jensen et al. [115] explained that organisations that are involved much earlier in the research process may better support vulnerable employees and establish practice-based research networks. Therefore, employers should invest in EBP tools and skills to create a culture of evidence-based supportive practices and a sustainable working environment for older employees with CMSDs.

### 4.9. The Role of the Manager in Supporting Older Employees

The integrated findings illustrated the importance of a supportive manager who could facilitate a flexible work environment, make adjustments at work and actively listen to employees’ specific needs. The importance of the manager’s role in promoting and maintaining the workforce’s health and wellbeing has already been highlighted in government documents and other initiatives discussed above. Making even small adjustments to a work role or setting can benefit employees with chronic conditions [116,117,118,119]. However, without OHS support, the responsibility for helping employees manage a CMSD rests predominantly with managers. The study also highlights the importance of open discussions about health matters, workplace adjustments and the design of realistic strategies that could reinforce employees’ own efforts to manage their condition at work. However, it also appears that managers do not, in general, understand the impact that CMSDs have on employees’ working lives. Open-plan offices may offer short-term financial benefits for the employer [120], but the findings revealed that new work strategies (e.g., agile working or hot-desking in open-plan offices) were mainly perceived negatively by the employees. Therefore, more research that evaluates the impact of the new working trends on the ageing workforce or employees with CMSDs is needed.

In addition, employees in this study perceived that managers were “at odds” with the government’s recommendations, mainly due to a lack of knowledge concerning laws and regulations. An underlying cause for this conflict may be the complexity and the ambiguity of the published guidelines, combined with the manager’s limited experience in dealing with the current changes in employment laws and policies [107,121]. Another important finding was that the manager was not always aware of the relevant resources, services or useful strategies that could be applied in the workplace. The manager’s role is essential, as they often are the “face of the employer or organisation” and they can act as a gatekeeper in directing employees to appropriate and available services. As organisations are currently adapting to the ageing workforce, it will be imperative for managers to re-imagine the profile of a “typical” employee and provide a range of alternative working arrangements as necessary.

### 4.10. The Role of Self-Management and Professional Health Services in Supporting Older Employees with CMSDs

Employees who participated in this study identified a variety of services that were appropriate and useful in supporting their own efforts to self-manage their condition in the workplace. Older employees with CMSDs frequently consulted healthcare professionals privately, as they required interventions that were individualised and took into account different environmental contexts and their specific needs. Previous research suggests that interventions offered by healthcare professionals who are trained to provide vocational support, such as physiotherapists and occupational therapists, can improve work participation and facilitate the management of CMSDs [122,123,124,125].

Older employees in this study were motivated to self-manage the condition, and most of them clearly understood the necessity of taking their health into their own hands. Interestingly, most primarily used passive strategies such as medication or massage to manage their condition. Some authors have suggested that these management choices indicate a desire for, or an expectation of, a “cure” or a “quick fix” requiring minimal self-contribution [126,127]. Our study findings add to the self-management literature and suggest that employees need more detailed information about how to maintain a healthy lifestyle approach, in order to support their sense of confidence and motivation in adopting and assimilating healthy choices into their lifestyle. Researchers have highlighted the role of self-management programmes [37,38] and the importance of prescribed exercises for those with a CMSD [39,40]. There is also evidence that occupational health advice is an essential element in designing an effective self-management programme [126,128]. Older employees with a CMSD should be encouraged to take an active approach if long-term self-management is to be achieved and work-related health maintained [128,129,130,131].

### 4.11. Strengths and Limitations of the Study

The authors O’Cathain [132], Heyvaert et al. [133] and Halcomb [134] addressed issues related to evaluating primary MMR studies and proposed that individual components of an MMR study should be evaluated for rigour in isolation. We were guided by their work in incorporating strategies aimed at increasing the rigour of both components of this study. Multiple strategies were used to ensure the rigour of the qualitative process and to ensure that the qualitative findings accurately reflected the participants’ experiences. The primary researcher consistently documented her own process during the study implementation and used these reflections to support the rigour of the analytic process. Similarly, the process by which the questionnaire was designed, constructed and administered enhanced the validity and reliability of the survey research phase. The data analysis approaches used in each phase followed systematic procedures. Elements of the study components were consistently reviewed by the supervisory committee members.

Reviews of the interview transcripts revealed some limitations related to the relative inexperience of the lead researcher in qualitative interviewing. Some issues raised by the participants may not have been fully explored, e.g., further probing questions could have been asked about the topic of leaveism. Similarly, personal characteristics, past experiences, gender, ethnicity and professional position may have influenced the participants’ interview responses, the probing questions and the interpretation of the data. However, different strategies were used to overcome these obstacles, for example by documenting in an online journal thoughts and feelings that could have influenced the methodological decisions made in the qualitative phase of this study. As the researchers were constrained by the timeframe they were operating within, it was difficult to determine how many interviews to conduct in different types of companies.

There were practical limitations in accessing potential respondents and administering the questionnaire in relevant workplace organisations which proved unavoidable. It was not possible to calculate the size of the target population (i.e., older employees with a CMSD in the West Midlands) from existing sources. In addition, we were unable to keep a record of how many questionnaires were administered (an issue frequently associated with online administered questionnaires), and therefore it was not possible to calculate a response rate (RR). This was a small exploratory MMR study with a clear aim and clear objectives and participant inclusion and exclusion criteria, focused on a limited number of diverse workplace organisations in the West Midlands. It was, therefore, not our intention to claim generalisability. It is our hope that by providing a detailed account of all aspects of the study, other researchers interested in a similar topic may be able to transfer these details to their own research setting and expand and build on our findings.

The study revealed the following implications for practice:

- Discrepancies exist between how employers, managers and supervisors interpret and implement current employment policies and strategies to support older employees in managing CMSDs at work.
- The social context of the workplace and the positive attitudes and understanding of colleagues and managers significantly contribute to how older employees view their future work ability and how they manage CMSDs in the workplace.
- The involvement of relevant healthcare professionals can make an important contribution to supporting work adjustments, translating best evidence into practice and assisting managers and employers to build an inclusive work environment and individualised strategies for employees with CMSDs.
- Research related to OHS provision should be developed that encompasses the impact on work of musculoskeletal health and the co-existence of multiple chronic conditions, particularly in relation to the ageing workforce and subgroups such as women.

### 4.12. Recommendations for Future Research

Future plans to assist older employees to manage CMSDs should be proactive in order to extend effects beyond the short-term management of the condition and consider how long-term musculoskeletal health can be optimised. Within the work environment, attention should be paid to the social wellbeing of older employees, and the uncertainty that they experience as they attempt to manage CMSDs at work should be acknowledged. Lastly, future strategies that take into account factors such as the complexity of the organisation, the diversity of the workforce and the managers’ abilities and responsibilities should be carefully implemented.

## 5. Conclusions

This study suggests that older employees with chronic musculoskeletal disorders require individualised support from employers and co-workers if they are to remain employable for as long as they need. Further support is needed, particularly for less well-recognised subgroups of the ageing workforce, e.g., women and those with chronic inflammatory disorders. Current challenges call for employers to focus on identifying effective ways to support the ageing workforce and invest in education and training opportunities for managers or collaborative opportunities with healthcare professionals and other stakeholders. This research also highlights that a flexible, empathetic and resourceful work environment is required, in order to support sustained employability for an ageing workforce.

## Figures and Tables

**Table 1:** Inclusion and exclusion criteria for the participants and respondents.

Inclusion Criteria	Exclusion Criteria
Working-age adults (>50 years)	Specific pathological conditions (e.g., tumours, infections, fractures). Hypertension or cardiovascular diseases, symptomatic disc prolapses or severe cervical spine disorders, postoperative conditions in the neck and shoulder region, history of severe physical trauma and pregnancy.
All types of jobs	Applied for an early retirement
Reported chronic conditions (12 weeks or more) involving any area of the body	Acute musculoskeletal conditions
Public or private sector	Self-employed or part-time
Ability to understand and speak English	Not able to understand and speak English

**Table 2:** Organisation of the online questionnaire into sections.

Section 1: Demographics
Section 2: Employment status
Section 3: Impact of the chronic musculoskeletal condition
Section 4: Management pathways
Section 5: Barriers towards the management of your condition
Section 6: Future plans and retirement

**Table 3:** An example of the narrative joint display for the theme “impact on wellness”.

Overarching Themes	Categories	Quantitative Findings	Qualitative Findings	Meta-Inferences and Interpretation	
Impact on wellness	Work performance	A total of 53% *(N* = 57/107) declared poor work ability, and 60% (*N* = 64/107) reported that the CMSD had interfered “quite a lot/extremely” with their ability to work effectively during the past six months.	I mean I ruined my hands working with no support for about 20 years. That is why I am worse now (Josh) I don’t know how much it really affects me now. I guess it affected me when I was having to go and have some physio a few times (before Christmas around 6 months ago). (Kathryn)	Convergence Expansion	Participants identified that work ability was affected at diverse levels. This was also confirmed by the quantitative responses. Both sets of findings demonstrated that work was affected in the past 6 months. Work ability was affected differently for the employees in the study. The condition fluctuates through the year due to the type of the CMSD, the use of medication, the job role they have and other factors affecting the intensity, e.g., stress, depression, comorbidities, flare-ups.

**Table 4:** Socio-demographic characteristics of interview participants.

Participant Pseudonyms	Age	Employment	Interview Duration	Current CMSD
Claire	58	Academia	70 min	Chronic pain
Debra	54	Mental health nurse practitioner	45 min	Scoliosis and chronic back pain
Sarah	52	City council (administrator)	58 min	Chronic hip pain
Nicky	55	Nurse practitioner	50 min	Chronic back pain
Teresa	57	Academia	45 min	Scoliosis and ankle pain
Anne	63	Customer service	75 min	Rheumatoid arthritis
Andrew	51	Mechanical engineer	52 min	Ankylosing spondylitis
Jessica	52	Human resources	50 min	Psoriatic arthritis
Kathryn	50	Accountant	40 min	Low back pain
John	62	Mechanical engineer	40 min	Arthritis
Laura	52	Travel agent	45 min	Chronic neck and shoulder pain
Maria	60	History teacher	42 min	Chronic back pain
Sally	51	Prevent education officer	79 min	Psoriatic arthritis
Annette	51	Laboratory technician	40 min	Arthritis
Jack	64	Design engineer	62 min	Ankylosing spondylitis
Josh	55	IT engineer	55 min	Arthritis

**Table 5:** Survey phase respondent socio-demographic characteristics (*N* = 107).

Gender	Count (*N*)	Percentage (%)
Male	44	41
Female	63	59
**Age group**		
50–55	52	49
56–60	36	33
60–65	17	16
>66	2	2
**Ethnicity**		
British	97	90
Other	10	10
**Work Location**		
West Midlands	81	76
Other	26	24

**Table 6:** Final categories that contributed to each qualitative theme.

Main Themes	Final Contributing Categories
Impact on wellness	Work performance
	Physical issues
	Mental stressors
	Personal life
Strategies and facilitators that support managing a CSMD	Taking a healthy approach
	Strategies offered at work
	Supportive environment
	Managing the condition outside the workplace
Perceived barriers related to management of a CMSD	Workstation design and environment
	Bureaucracy and procedures
	Unsupportive colleagues
	Barriers with the management team
	Healthcare system
Employees’ approach to living with a CMSD	Work ethic
	Attitudes to management strategies
	Taking responsibility for self-management
	Disclosing the condition
Emotions and beliefs about future employment and retirement	Fear of employment
	Motivation to work longer
	Government changes and healthy retirement

## Data Availability

Not applicable.

## Supplementary Materials

The following supporting information can be downloaded at: https://www.mdpi.com/article/10.3390/ijerph19159348/s1.
