# Supplementary material for: Management of Chronic Musculoskeletal Disorders in the Workplace from the Perspective of Older Employees: A Mixed Methods Research Study
Source: Int J Environ Res Public Health. 2022 Jul 30;19(15):9348. doi: 10.3390/ijerph19159348 (PMC9367967; doi:10.3390/ijerph19159348)
Supplement: Supplementary file 1 [file ijerph-19-09348-s001.zip › ijerph-1779500-supplementary.pdf]

Page 2: Section S1: Demographics

These are some demographic questions that will be used only for classification.

What is your age group?

- ◌ 50-55
- ◌ 56-60
- ◌ 61-65
- ◌ 66-70

Please fill in your gender

- ◌ Male
- ◌ Female
- ◌ Prefer not to say

Were you born in the UK?

- ◌ Yes
- ◌ No

Do you work in **Midlands?**

- ◌ Yes
- ◉ Other

If you selected Other, please specify:

In a few words, can you describe the main chronic musculoskeletal disorder (MSD) you have?

And how long have you had this musculoskeletal disorder?

- ◌ Less than 3 months
- ◌ 3-6 months
- ◌ More than 6 months
- ◌ More than 2 years

Page 3: Section S2: Employment

Which of the following best describes your present work situation? (Please choose only one)

- Employed Full-time
- Employed Part-time
- Retired

What is your job title?

In what industry do you work? (Please check the category that apply to you)

- Agriculture, Forestry, Fishing
- Mining
- Manufacturing
- Electricity, Gas, Water, Waste Services
- Construction
- Retail Trade
- Transport, Postal and Warehousing
- Financial and Insurance Services
- Professional, Scientific, Technical Services
- Administrative and Support Services
- Education and Training
- Health Care and Social Assistance
- Other Services
- Other

If you selected Other, please specify:

How long have you been employed at your current company?

- Less than a year
- 1-5 years
- More than 5 years

How many people in total work for your employer?

- 1-9
- 10-29
- 30-99
- 100-500
- More than 500

In your main job, does an average day at work involve any of the following activities? (Please check all categories that apply to you)

- ☐ Kneeling or squatting for longer than 1 hour per day in total
- ☐ Climbing a ladder
- ☐ Climbing up and down more than 30 flights of stairs per day
- ☐ Lifting weights of 10 kg or more
- ☐ Lifting weights of 2-10 kg
- ☐ Standing or walking for most of the day
- ☐ Standing or walking for more than 3 hours at a time
- ☐ Sitting for most of the day
- ☐ Driving for most of the day
- ☐ Driving towards different sites
- ☐ Driving between cities
- ☐ Repetitive or awkward movements such as twisting or bending
- ☐ Tasks that require intense concentration
- ☐ Other

If you selected Other, please specify:

Page 4: Section S3: Impact of your chronic musculoskeletal condition

How important it is for you to perform well in these different aspects of your life? (Please select one answer per row)

	(1) Not at all Important	(2) Not very Important	(3) Fairly Important	(4) Very Important	(5) Extremely Important
Job Role and work responsibilities	☐	☐	☐	☐	☐
Social activities and hobbies	☐	☐	☐	☐	☐
Physical activities and sports	☐	☐	☐	☐	☐
Emotional wellbeing	☐	☐	☐	☐	☐
Personal life and responsibilities	☐	☐	☐	☐	☐
Finances and future plans	☐	☐	☐	☐	☐

Assume that your work ability at its best has a value of 10 points. How many points would you give your current work ability? (0 means that you cannot currently work at all)

	0	1	2	3	4	5	6	7	8	9	10	
Completely unable to work	☐	☐	☐	☐	☐	☐	☐	☐	☐	☐	☐	Best work ability

How much did your condition interfere with your ability to work in the last 6 months?

- ○ Not at all
- ○ A little bit
- ○ Moderately
- ○ Quite a bit
- ○ Extremely

In relation to your job role and responsibilities, how much does your health condition affect your ability to perform work tasks? (Tick at least one)

	(1) Not at all	(2) A little bit	(3) Moderately	(4) Quite a bit	(5) Extremely	Not applicable
Kneeling or squatting	☐	☐	☐	☐	☐	☐
Twisting or bending forward	☐	☐	☐	☐	☐	☐
Lifting weight	☐	☐	☐	☐	☐	☐
Standing	☐	☐	☐	☐	☐	☐
Opening jars	☐	☐	☐	☐	☐	☐
Walking	☐	☐	☐	☐	☐	☐
Sitting for prolonged period of time	☐	☐	☐	☐	☐	☐
Driving	☐	☐	☐	☐	☐	☐
Going up/down the stairs	☐	☐	☐	☐	☐	☐
Other: Work task	☐	☐	☐	☐	☐	☐

If you selected Other, please specify the task(s):

Page 5: Section S4: Management Pathways

Who at work do you mostly talk about your condition? ✱ *Required*

- ☐ Colleagues
- ☐ Supervisor
- ☐ Line manager
- ☐ Employer
- ☐ Other 1
- ☐ Other 2

If you selected Other, please specify:

How often do you get support at work from the following individuals?

	(1) Almost Never	(2) Seldom	(3) Sometimes	(4) Often	(5) Almost Always	Not applicable
Colleagues	☐	☐	☐	☐	☐	☐
Supervisor	☐	☐	☐	☐	☐	☐
Line manager	☐	☐	☐	☐	☐	☐
Employer	☐	☐	☐	☐	☐	☐

What strategies have you been offered in the workplace to help you manage your condition? (Please check all categories that apply to you)

- ☐ Flexible working hours
- ☐ Working from home
- ☐ Refer to occupational health
- ☐ Provide Private healthcare
- ☐ Exercise classes
- ☐ Refer to on site physiotherapy services
- ☐ Online ergonomics assessments
- ☐ Other

If you selected Other, please specify:

Please specify any stategies you were offered in your **previous** workplace (if applicable) *Optional*

How **effective** were the strategies offered to help you manage your condition in the workplace? (Please check **at least one that applies to you**)

	(1) Not at All Effective	(2) Not Very Effective	(3) Somewhat Effective	(4) Very Effective	(5) Extremely effective	Not used/Not provided
Flexible working hours	☐	☐	☐	☐	☐	☐
Working from home	☐	☐	☐	☐	☐	☐
Refer to occupational health	☐	☐	☐	☐	☐	☐
Provide Private healthcare	☐	☐	☐	☐	☐	☐
Onsite gym/exercise classes	☐	☐	☐	☐	☐	☐
Refer to on-site physiotherapy services	☐	☐	☐	☐	☐	☐
Online ergonomics assessments	☐	☐	☐	☐	☐	☐
Other	☐	☐	☐	☐	☐	☐

If you selected Other, please specify:

How important are these strategies in helping you manage your condition? (Please select **at least one that applies to you**)

	(1) Not at All Important	(2) Not Very Important	(3) Fairly Important	(4) Very Important	(5) Extremely Important	Not applicable
Flexible working hours	☐	☐	☐	☐	☐	☐
Working from home	☐	☐	☐	☐	☐	☐
Refer to occupational health	☐	☐	☐	☐	☐	☐
Provide Private healthcare	☐	☐	☐	☐	☐	☐
Onsite gym/exercise classes	☐	☐	☐	☐	☐	☐
Refer to on-site physiotherapy services	☐	☐	☐	☐	☐	☐
Online ergonomics assessments	☐	☐	☐	☐	☐	☐
Other	☐	☐	☐	☐	☐	☐

If you selected Other, please specify:

How **do you** manage your chronic MSD at the workplace? (Please check all categories that apply to you) ✱ *Required*

- ☐ Take regular breaks
- ☐ Reduced working hours
- ☐ Use of ergonomic equipment
- ☐ Medication
- ☐ Lifestyle changes
- ☐ Meditation, Mindfulness etc.
- ☐ On site gym/exercise classes
- ☐ Visit on site healthcare professionals
- ☐ Monitor physical activity (e.g. wearable watch, phone apps)
- ☐ Other

If you selected Other, please specify:

If you selected "Lifestyle changes", please specify *Optional*

Have you ever used any of the following strategies to help you manage your condition? (Select all that apply)

- ☐ Physiotherapy
- ☐ Sports therapy
- ☐ Massage
- ☐ Acupuncture
- ☐ Exercises
- ☐ Pilates
- ☐ Yoga
- ☐ Aromatherapy
- ☐ Swimming pool
- ☐ Ergonomic pillow
- ☐ Ergonomic chair
- ☐ Standing up desk
- ☐ Splints
- ☐ Vitamins and supplements
- ☐ Other

If you selected Other, please specify: *Optional*

How important are these strategies for the management of your condition? (Please check **at least one**)

	(1) Not at All Important	(2) Not Very Important	(3) Fairly Important	(4) Very Important	(5) Extremely Important	Not used/Not applicable
Take regular breaks	☐	☐	☐	☐	☐	☐
Reduced working hours	☐	☐	☐	☐	☐	☐
Use of ergonomic equipment	☐	☐	☐	☐	☐	☐
Medication	☐	☐	☐	☐	☐	☐
Lifestyle changes	☐	☐	☐	☐	☐	☐
Psychological techniques	☐	☐	☐	☐	☐	☐
See on-site healthcare professional	☐	☐	☐	☐	☐	☐
Use of gym/exercise classes	☐	☐	☐	☐	☐	☐
Other	☐	☐	☐	☐	☐	☐

If you selected Other, please specify

We would like to know how satisfied you are with the support you get at work. Please indicate the degree of agreement with the statements below

	(1) Strongly Disagree	(2) Disagree	(3) Neutral	(4) Agree	(5) Strongly Agree	Not applicable
My employer supports me to manage my condition at the workplace	☐	☐	☐	☐	☐	☐
My line manager supports me to manage my condition at the workplace	☐	☐	☐	☐	☐	☐
I am satisfied with the strategies offered at my workplace to manage my condition	☐	☐	☐	☐	☐	☐
My colleagues do not recognise/understand my condition	☐	☐	☐	☐	☐	☐
I am aware of the support I can get at the workplace	☐	☐	☐	☐	☐	☐
My line manager is aware of my condition	☐	☐	☐	☐	☐	☐
My employer is aware of my condition	☐	☐	☐	☐	☐	☐
My colleagues are helpful and supportive when I am not feeling well	☐	☐	☐	☐	☐	☐
My employer does not recognise/understand my condition	☐	☐	☐	☐	☐	☐
My line manager does not recognise/understand my condition	☐	☐	☐	☐	☐	☐

What strategies would you liked to have been offered at the workplace? *Optional*

Page 6: Section S5: Barriers

We would like to know your thoughts about some factors that can affect how you manage your condition at work. Please indicate the degree of agreement with the statements below. (Please check **at least one**)

	(1) Strongly Disagree	(2) Disagree	(3) Neutral	(4) Agree	(5) Strongly Agree	Not applicable
My workstation requires alterations to accommodate my needs	☐	☐	☐	☐	☐	☐
I have discussed my needs with the Occupational Health team at my workplace	☐	☐	☐	☐	☐	☐
I do not like others to know about my condition	☐	☐	☐	☐	☐	☐
I know what is offered at the workplace to manage my condition	☐	☐	☐	☐	☐	☐
Managing my condition at the workplace can reduce the burden on the NHS	☐	☐	☐	☐	☐	☐
I have access to occupational health or other health professionals at my workplace	☐	☐	☐	☐	☐	☐
People at work are well-educated about chronic musculoskeletal conditions	☐	☐	☐	☐	☐	☐
I believe that my organisation values the employees	☐	☐	☐	☐	☐	☐
I have quick access to different healthcare professionals through the NHS	☐	☐	☐	☐	☐	☐
It is very important to self-manage my condition	☐	☐	☐	☐	☐	☐

Similarly, please indicate how often the following occur.

	(1) Almost Never	(2) Seldom	(3) Sometimes	(4) Often	(5) Almost Always	Not applicable
On a normal day I work with pain or discomfort	☐	☐	☐	☐	☐	☐
I take sick leave when I am not feeling well	☐	☐	☐	☐	☐	☐
I ask my colleagues to help me with a task I cannot perform at work	☐	☐	☐	☐	☐	☐
I would pay privately to see a healthcare professional	☐	☐	☐	☐	☐	☐
I find time to self-manage my condition	☐	☐	☐	☐	☐	☐
I stay at work even on the days i feel unwell	☐	☐	☐	☐	☐	☐
My condition affects my work abilities and performance	☐	☐	☐	☐	☐	☐
I consult my local GP to discuss my condition	☐	☐	☐	☐	☐	☐

Please indicate how much you agree with the statements below.(Please check **at least one**)

	(1) Strongly Disagree	(2) Disagree	(3) Neutral	(4) Agree	(5) Strongly Agree	Not applicable
My condition makes me depressed (feeling sad)	☐	☐	☐	☐	☐	☐
I am happy with the support I get from my organisation	☐	☐	☐	☐	☐	☐
I feel confident that older employees with chronic MSDs are well supported	☐	☐	☐	☐	☐	☐
I am afraid to reveal my condition to my employer	☐	☐	☐	☐	☐	☐
I am happy with the support I get from the NHS	☐	☐	☐	☐	☐	☐
I am confident that I can manage my condition well	☐	☐	☐	☐	☐	☐
I feel hopeless about my future due to my condition	☐	☐	☐	☐	☐	☐
I feel frustrated when my colleagues do not understand my needs	☐	☐	☐	☐	☐	☐
I feel confident to discuss my needs with my line manager	☐	☐	☐	☐	☐	☐
I feel frustrated when I cannot perform well at my job	☐	☐	☐	☐	☐	☐

Page 7: Section S6: Future Plans

The Retirement age and the State Pension Age has changed for both men and women. Although there is no longer a law that states that you have to retire at a certain age, the Government is planning further increases of the State Pension Age from 66 to 68 years. Please indicate how much you agree with the statements below.

	(1) Strongly Disagree	(2) Disagree	(3) Neutral	(4) Agree	(5) Strongly Agree	Not applicable
I am adequately informed about the pension age changes	☐	☐	☐	☐	☐	☐
I feel confident that I can work until my State Pension Age	☐	☐	☐	☐	☐	☐
Changes to the State Pension Age would impact my ability to enjoy retirement	☐	☐	☐	☐	☐	☐
I have a plan of how I will manage my condition until I retire	☐	☐	☐	☐	☐	☐
I am worried about my future employment due to my condition	☐	☐	☐	☐	☐	☐
I am confident that I can manage my finances well	☐	☐	☐	☐	☐	☐

Do you expect to carry out the same type of work until you retire? ✱ *Required*

- ○ Yes
- ○ No
- ○ Not Sure

Can you briefly explain your answer? *Optional*

____

Is there anything else you would like to add?

____
